# Supplementary material for: A Novel Benzopyrane Derivative Targeting Cancer Cell Metabolic and Survival Pathways
Source: Cancers (Basel). 2021 Jun 7;13(11):2840. doi: 10.3390/cancers13112840 (PMC8201054; doi:10.3390/cancers13112840)
Supplement: Supplementary file 1 [file cancers-13-02840-s001.zip › supplementary material/figure S1-S11.pdf]

A Novel benzopyrane derivative targeting cancer cell metabolic and survival pathways

Dana M. Zaher1,2,#, Wafaa S. Ramadan1,2,#, Raafat El-Awady1,3, Hany A. Omar1,3,4, Fatema Hersi1,2, Vunnam Srinivasulu1, Ibrahim Y. Hachim1,2, Farah I. Almarzooq1,5, Cijo G. Vazhappilly1,6, Salim Merali7, Carmen Merali7, Nelson C. Soares1,3, Paul Schilf 8, Saleh M. Ibrahim1,2,8, Taleb H. Al-Tel1,3,*

[1]Sharjah Institute for Medical Researches, University of Sharjah, Sharjah, United Arab Emirates.

[2]College of Medicine, University of Sharjah, Sharjah, United Arab Emirates.

[3]College of Pharmacy, University of Sharjah, Sharjah, United Arab Emirates.

[4]Faculty of Pharmacy, Beni-Suef University, Beni-Suef, 62511 Egypt

[5]United Arab Emirates University, Department of Medical Microbiology and Immunology, College of Medicine and Health Sciences, Al Ain, United Arab Emirates.

[6]School of Arts and Sciences, American University of Ras Al Khaimah, P.O.Box:10021, Ras Al Khaimah, United Arab Emirates.

[7]Temple University, School of Pharmacy, 3307 N Broad Street, Room 552, Philadelphia, PA 19140, United States.

[8]Lübeck Institute of Experimental Dermatology, University of Lübeck, Ratzeburger Allee 160 23538 Lübeck, Germany.

*Correspondance: taltal@sharjah.ac.ae, Tel: +97165057417

# These coauthors contributed equally to this work and are both first authors

Supplementary Figure S1: Screening of a small library of polysubstituted benzopyranes, for potential anti-cancer activity on cancer cell lines. **(a)** Dose dependent effect of the synthesized SIMR compounds on MCF7, SKBR3 and HCT-116 after 48 h treatment, as analyzed by SRB assay. Points, mean; bars, SEM (n=6). **(b)** The IC_{50} of SIMR compounds in cancer cell lines after 48 h treatment. Data are mean ± SEM (*n=3)*. **(c)** Dose dependent effect of the synthesized SIMR -1281 on MCF7, MCF7 dox resistant, A549 and A549 dox resistant cell lines after 48 h treatment, as analyzed by MTT assay. Points, mean; bars, SEM (*n=6*).

a

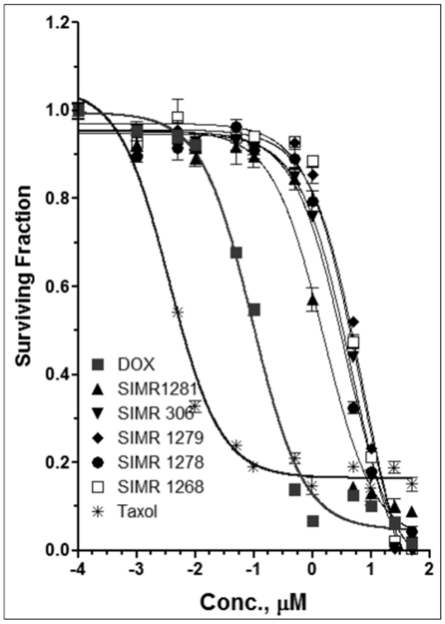

b

Compound	IC50(μM)		
	HCT116	MCF7	SKBR3
DOX	0.096	0.058	0.25
SIMR1281	1.554	1.91	1.9
SIMR306	4.739	11.8	9.4
SIMR1279	7.217	8.14	7.6
SIMR1278	3.733	4.5	6.4
SIMR1268	6.264	6.76	10.1

c

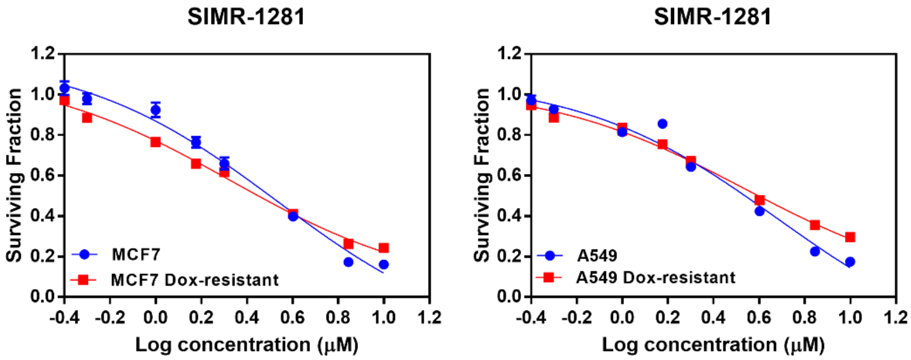

Supplementary Figure S2: DARST Assay showing the biding of SIMR1281 with protein targets

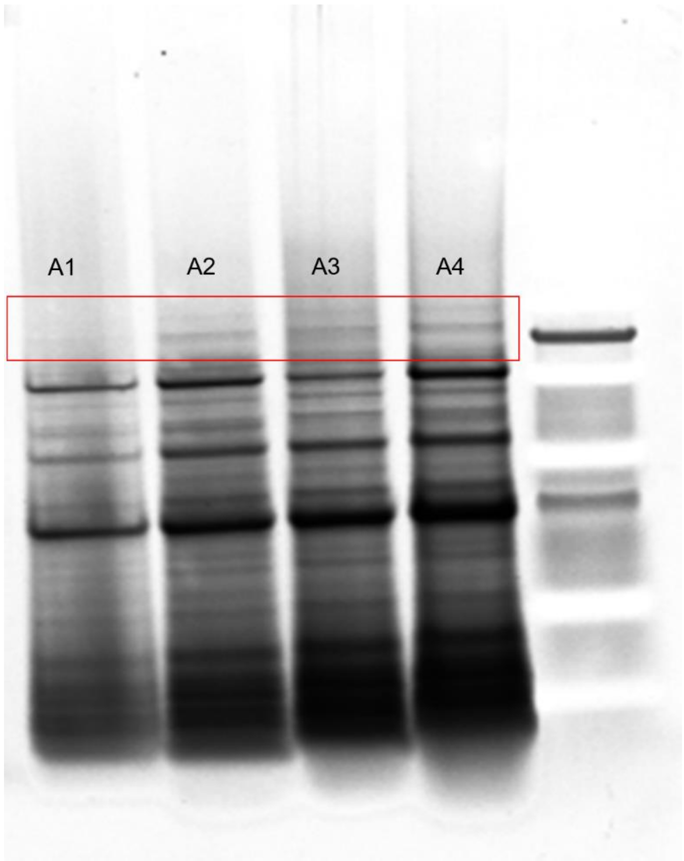

Supplementary Figure S3: Western blot analysis for γ-H2Ax/H2Ax, p-ATM/ATM and p-ATR/ATR proteins in MCF7, HCT116 and SKBR-3 cell lines after the treatment with SIMR-1281 and Dox for 24 h at the indicated concentrations (Whole blot corresponds to Fig3. a). **(a)** Quantification of the bands of γ-H2Ax, H2Ax, p-ATM, ATM, p-ATR and ATR proteins using Image lab software, Data expressed as mean ± SEM (*n=3*) independent experiments. *indicates significant difference versus control at p<0.05 determined by two-tailed unpaired *t*-test. **(b)** Full blot Western blot of the indicated proteins.

a

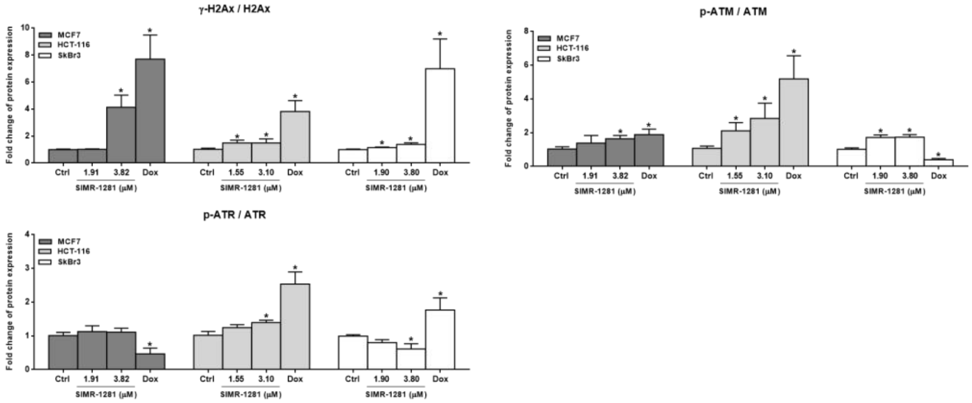

b

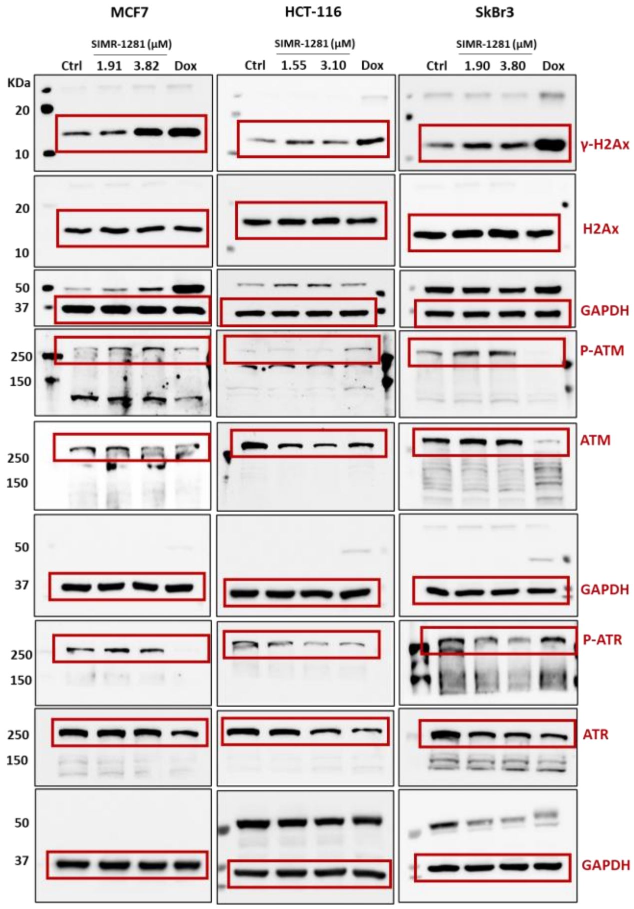

Supplementary Figure S4: Western blot analysis for p53, p21, p-Chk2/Chk2 and p-Chk1/Chk1 proteins in MCF7, HCT116 and SKBR-3 cell lines after the treatment with SIMR-1281 and Dox for 24 h at the indicated concentrations (Whole blot corresponds to Fig3. b). **(a)** Quantification of the bands of p53, p21, p-Chk2, Chk2, p-Chk1 and Chk1 proteins using Image lab software, Data expressed as mean ± SEM (*n=3*) independent experiments. *indicates significant difference versus control at p<0.05 determined by two-tailed unpaired *t*-test. **(b)** Full blot Western blot of the indicated proteins

a

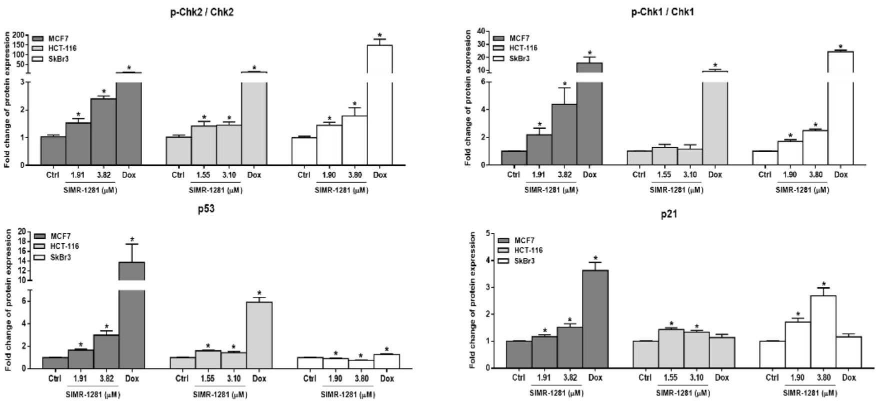

b

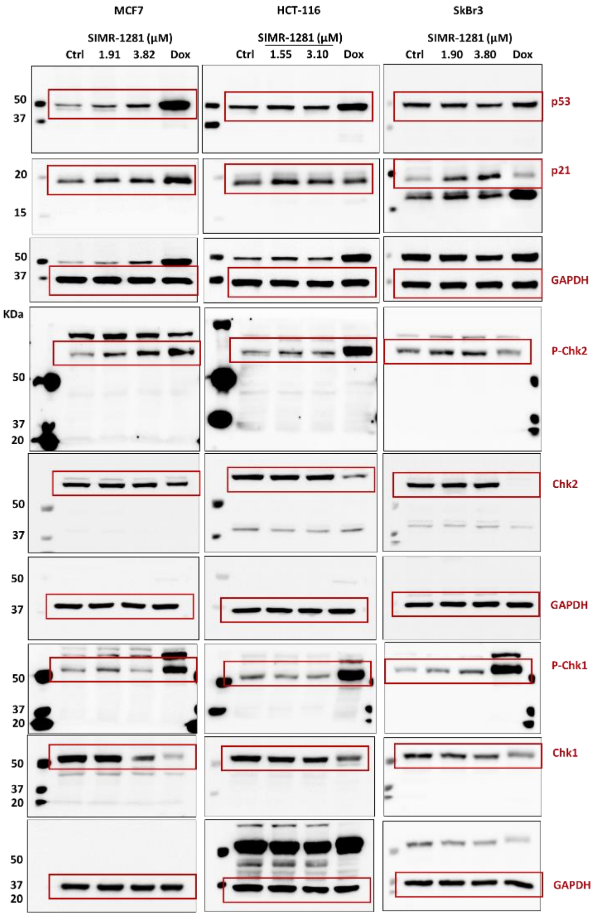

Supplementary Figure S5: Western blot analysis for Ras, p-ERK1/2, ERK1/2, p-Akt, Akt proteins in MCF7, HCT116 and SKBR-3 cell lines after the treatment with SIMR-1281 and Dox for 24 h at the indicated concentrations (Whole blot corresponds to Fig3. c). **(a)** Quantification of the bands of Ras, p-ERK1/2, ERK1/2, p-Akt, Akt proteins using Image lab software, Data expressed as mean ± SEM (*n=3*) independent experiments. *indicates significant difference versus control at p<0.05 determined by two-tailed unpaired *t*-test. **(b)** Full blot Western blot of the indicated proteins

a

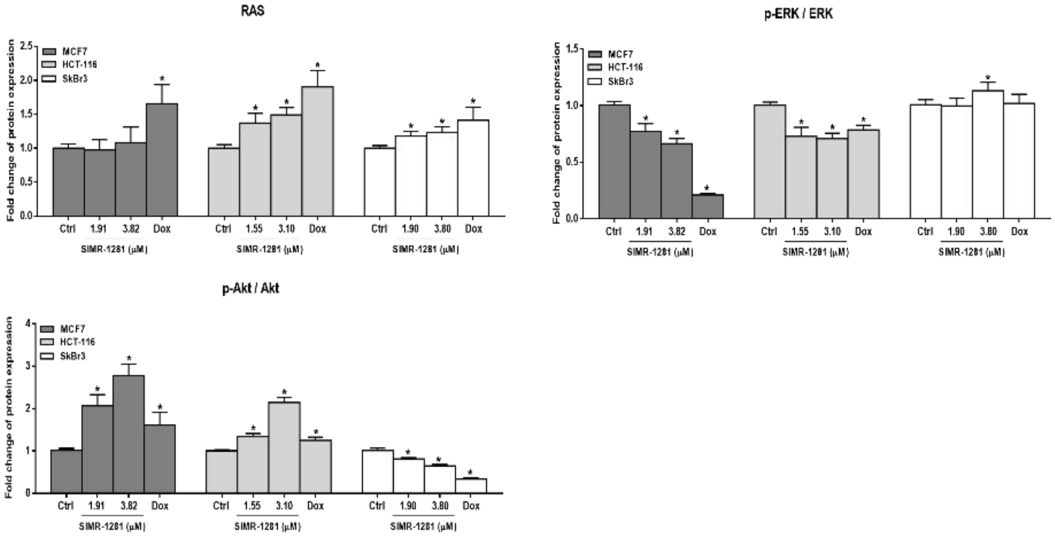

b

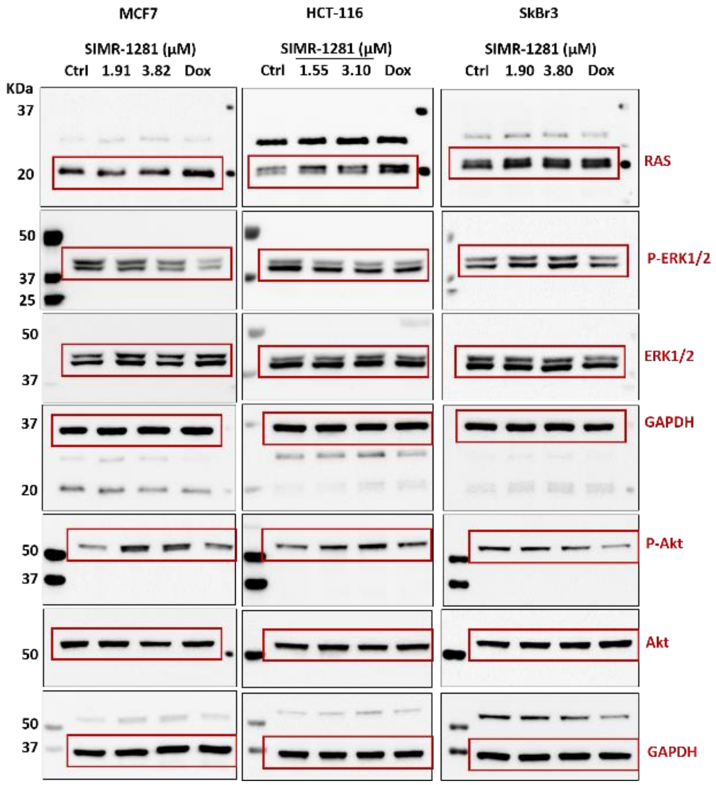

Supplementary Figure S6: Cell cycle analysis of MCF7, HCT116 and F-180 cells treated with SIMR-1281 at the indicated time points. (**a**) Histogram representation of the cell cycle distributions of MCF7, HCT116 and F-180 cells treated with SIMR-1281.

a

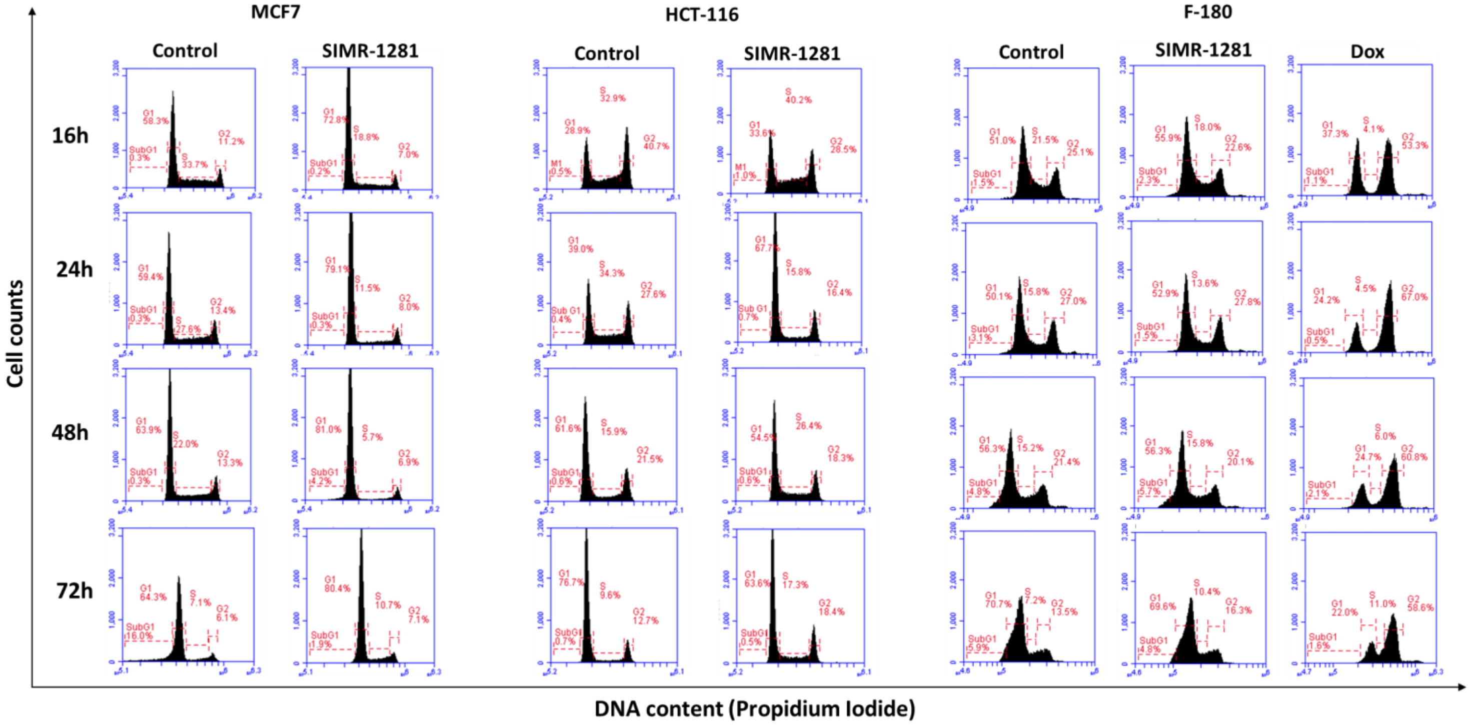

Supplementary Figure S7: Western blot analysis for caspase-3 and caspase-9 proteins in MCF7, HCT116 and SKBR-3 cell lines after the treatment with SIMR-1281 for 24 h at the indicated concentrations (Whole blot corresponds to Fig3. e). **(a)** Quantification of the cleavage bands of caspase 3 and caspase 9 proteins using Image lab software, Data expressed as mean ± SEM (*n=3*) independent experiments. *indicates significant difference versus control at p<0.05 determined by two-tailed unpaired *t*-test. **(b)** Full blot Western blot of the caspases proteins

a

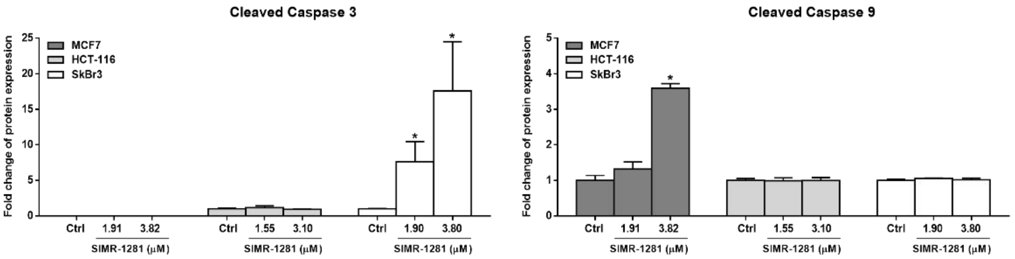

b

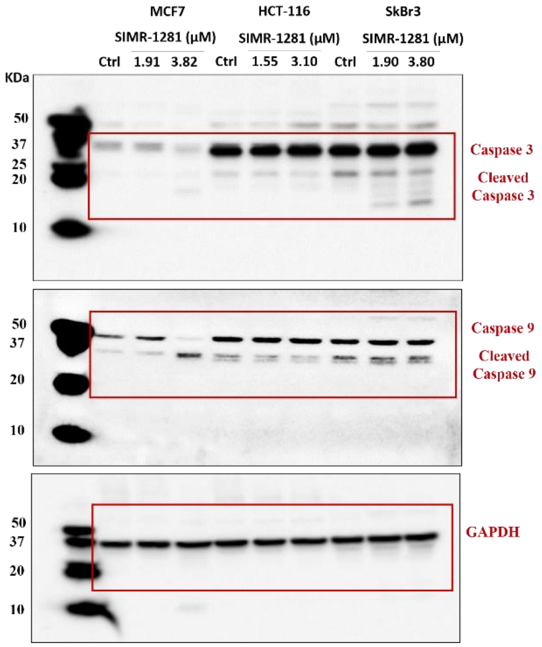

Supplementary Figure S8: Western blot analysis for apoptotic marker proteins in MCF7, HCT116 and SKBR-3 cell lines after the treatment with SIMR-1281 and Dox for 24 h at the indicated concentrations (Whole blot corresponds to Fig3. f). **(a)** Quantification of the apoptosis markers proteins bands using Image lab software, Data expressed as mean ± SEM (*n=3*) independent experiments. *indicates significant difference versus control at $p<0.05$ determined by two-tailed unpaired *t*-test. **(b)** Full blot Western blot of the apoptosis markers proteins

a

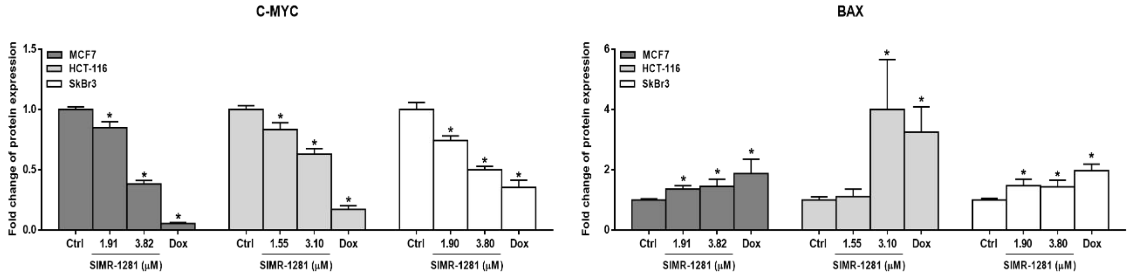

b

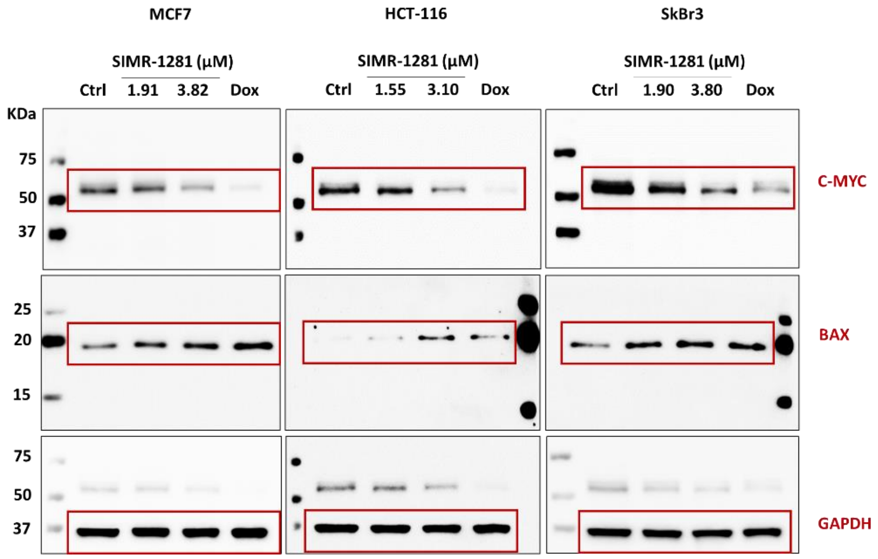

Supplementary Figure S9: Western blot analysis for cyclin A and cyclin B proteins in MCF7 cell line after the treatment with SIMR-1281 for 24 h at the indicated concentrations, (Whole blot corresponds to Fig4. c). **(a)** Quantification of cyclin A and cyclin B proteins using Image lab software, Data expressed as mean ± SEM (*n=3*) independent experiments. *indicates significant difference versus control at p<0.05 determined by two-tailed unpaired *t*-test. **(b)** Full blot Western blot of the cyclins proteins.

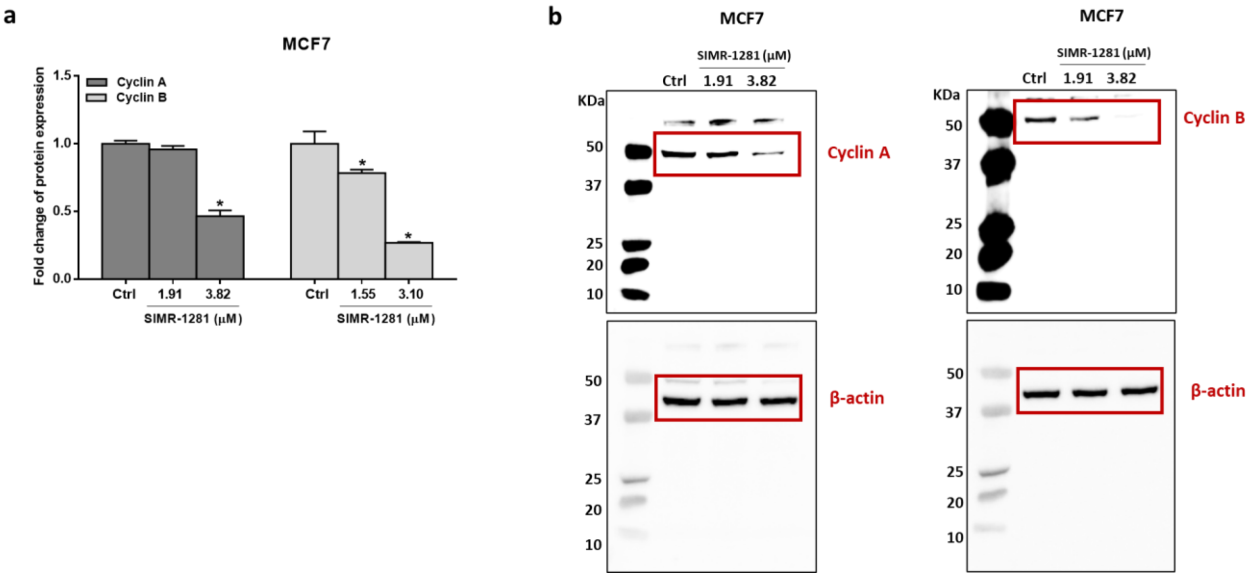

Supplementary Figure S10: Histogram representation of the cell cycle distributions of synchronized MCF7 cells at different time points.

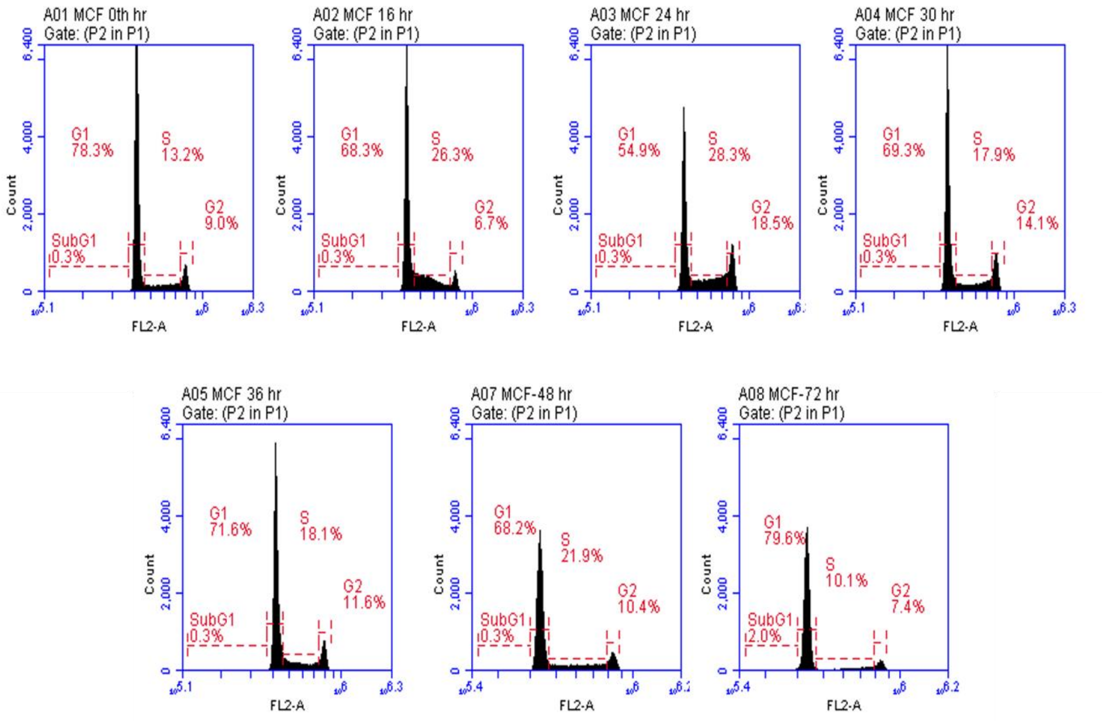

Supplementary Figure S11: Hematology values[a] and serum chemistry values[a] from athymic nude mice bearing subcutaneous HCT116 xenograft tumors after 30 days of treatment with SIMR-1281. Values represent means ± SD. [a] WBC, white blood cells; RBC, red blood cells; HCT, Hematocrit; MCV, mean corpuscular volume; MCH, Mean Corpuscular Hemoglobin; MCHC, Mean Corpuscular Hemoglobin Concentration; RDW, Red blood cell distribution width; RDWSD, Red blood cell distribution width; PLT, platelet; MPV, mean platelet volume; LY, leukocytes; MO, monocytes; NE, neutrophils; EO, eosinophils; BA, basophils. [B] ALT, Alanine aminotransferase; Aspartate Aminotransferase (AST); Alkaline Phosphatase (Alk Phos, ALP); Gamma Glutamyl Transpeptidase (GGT, GGTP or GTP); Significant difference versus control * P<0.05 **P<0.005 determined by two-tailed unpaired *t*-test.

Hematology[b]	Units	Vehicle	SIMR-1281 (50mg/kg)
WBC	x10^3/µl	2.44±0.85	2.22±1.15
RBC	x10^3/µl	7.18±0.31	6.72±0.45
Hemoglobin	g/dL	12.42±0.75	11.53±0.76
HCT	%	32.34±2.11	30.89±2.18
MCV	fL	44.99±1.35	45.96±1.10
MCH	pg	17.28±0.35	17.16±0.25
MCHC	g/dL	38.42±0.58	37.36±1.06
RDW	%	23.61±1.26	26.04*±1.43
RDWSD	fL	26.14±0.5	29.46*±3.12
PLT	x10^3/µl	853.63±95.68	718.09±371.26
MPV	fL	6.30±0.40	7.69±1.96
LY	%	90.28±0.40	86.64±7.46
MO	%	5.68±2.81	8.03±6.73
NE	%	3.16±1.72	4.25±1.77
EO	%	0.52±0.27	0.81±0.57
BA	%	0.36±0.21	0.27±0.14
**Serum Chemistry[c]**			
Creatinine	mg/dL	1.04±0.89	1.176±0.398
ALT	iu/L	25.19±5.55	44.35±14.84
AST	iu/L	91.25±20.50	180.72±54.93
ALP	iu/L	33.42±19.21	25.722±13.25
GAMMA GT		11.88±9.20	16.45±2.041
Total Bilirubin	mg/dL	0.548±0.040	0.7±0.327
Total Protein	g/dL	7.136±0.157	10.124±4.383
Albumin	g/dL	3.277±0.168	2.660**±0.2478
Urea		51.22±2.469	57.094±12.29
Glucose	mg/dL	283.55±20.00	176.59±50.93

.
