# Supplementary material for: A Novel Benzopyrane Derivative Targeting Cancer Cell Metabolic and Survival Pathways
Source: Cancers (Basel). 2021 Jun 7;13(11):2840. doi: 10.3390/cancers13112840 (PMC8201054; doi:10.3390/cancers13112840)
Supplement: Supplementary file 1 [file cancers-13-02840-s001.zip › supplementary material/file S1.pdf]

A Novel benzopyrane derivative targeting cancer cell metabolic and survival pathways

Dana M. Zaher[1,2,#], Wafaa S. Ramadan[1,2,#], Raafat El-Awady[1,3], Hany A. Omar[1,3,4], Fatema Hersi[1,2], Vunnam Srinivasulu[1], Ibrahim Y. Hachim[1,2], Farah I. Almarzooq[1,5], Cijo G. Vazhappilly[1,6], Salim Merali[7], Carmen Merali[7], Nelson C. Soares[1,3], Paul Schilf [8], Saleh M. Ibrahim[1,2,8], Taleb H. Al-Tel[1,3,*]

*Correspondance: taltal@sharjah.ac.ae, Tel: +97165057417

# These coauthors contributed equally to this work and are both first authors

Supplementary file S1

General: Chemical reagents and anhydrous solvents were purchased from Sigma-Aldrich and were used without further purification. TLC analysis was performed with silica gel plates (0.25 mm, E. Merck, 60 F_{254}) using iodine and a UV lamp for visualization. ^{1}H and ^{13}C NMR experiments were performed on a 500 MHz instrument. Chemical shifts are reported in parts per million (ppm) downstream from the internal tetramethylsilane standard. Spin multiplicities are described as s (singlet), d (doublet), dd (double doublets), t (triplet), (td) triple doublets or m (multiplet). Coupling constants are reported in Hertz (Hz). ESI mass spectrometry was performed on a Q-TOF high-resolution mass spectrometer or Q-TOF Ultim LC-MS. HPLC analysis was performed on Shimadzu Nexera-i LC-2040C HPLC with PDA detector, using a Restek Ultra AQ C18, 5 µm, 150 mm × 4.6 mm column, eluted using 0.1 % TFA in water and acetonitrile at 1.0 mL/min and detected at 220 nm.

Reaction procedure for the preparation of (8aS,14aS)-9,9-dimethyl-11,12-diphenyl-8a,14a-dihydro-8H,9H-benzo[5,6]chromeno[4,3-d]thiazolo[3,2-a]pyrimidine (SIMR-1281):

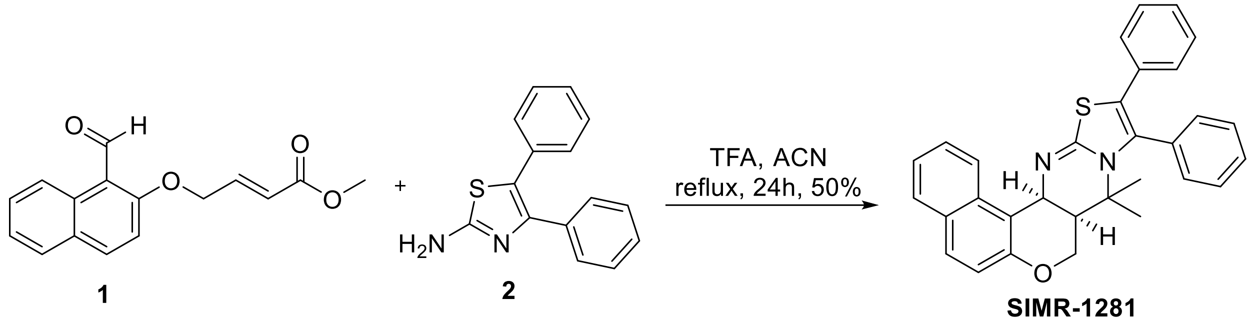

Methyl (E)-4-((1-formylnaphthalen-2-yl)oxy)but-2-enoate (**1**, 0.6 mmol) and 4,5-diphenylthiazol-2-amine (**2**, 0.5 mmol) were mixed in ACN (5 mL) at rt. Then, TFA (1.0 mmol) was added dropwise and refluxed for 24h. After completion of the reaction, ACN and excess TFA was removed under vacuum and concentrated to dryness. The crude material was triturated using a mixture of EtOAc and diethyl ether in different ratios to get SIMR-128 as a white solid. 118 mg (50% yield). ^{1}H NMR (500 MHz, $CDCl_3$) δ 8.19 (d, *J* = 8.4 Hz, 1H), 7.79 (t, *J* = 7.6 Hz, 2H), 7.68 (t, *J* = 7.5 Hz, 1H), 7.58 – 7.52 (m, 3H), 7.44 (dd, *J* = 9.7, 5.4 Hz, 2H), 7.33 (d, *J* = 7.6 Hz, 1H), 7.21 (dt, *J* = 22.4, 7.2 Hz, 3H), 7.06 (d, *J* = 9.0 Hz, 1H), 7.01 (d, *J* = 7.2 Hz, 2H), 5.51 (d, *J* = 3.1 Hz, 1H), 4.51 (dd, *J* = 10.8, 2.2 Hz, 1H), 4.09 (t, *J* = 11.5 Hz, 1H), 2.33 (dt, *J* = 12.0, 3.5 Hz, 1H), 1.89 (s, 3H), 1.20 (s, 3H). ^{13}C NMR (125 MHz, $CDCl_3$) δ 162.5, 151.8, 134.2, 132.5, 132.2, 131.7, 131.5, 130.8, 130.3, 129.3, 129.1, 128.9, 128.7, 128.6, 128.0, 124.5, 122.3, 121.7, 117.8, 117.7, 109.1, 61.55, 61.10, 43.62, 41.23, 30.81, 27.05. HRMS (ESI-TOF): m/z calcd for $C_{31}H_{27}N_2OS$ 475.1844, found 475.1004 $[M+H]^+$.

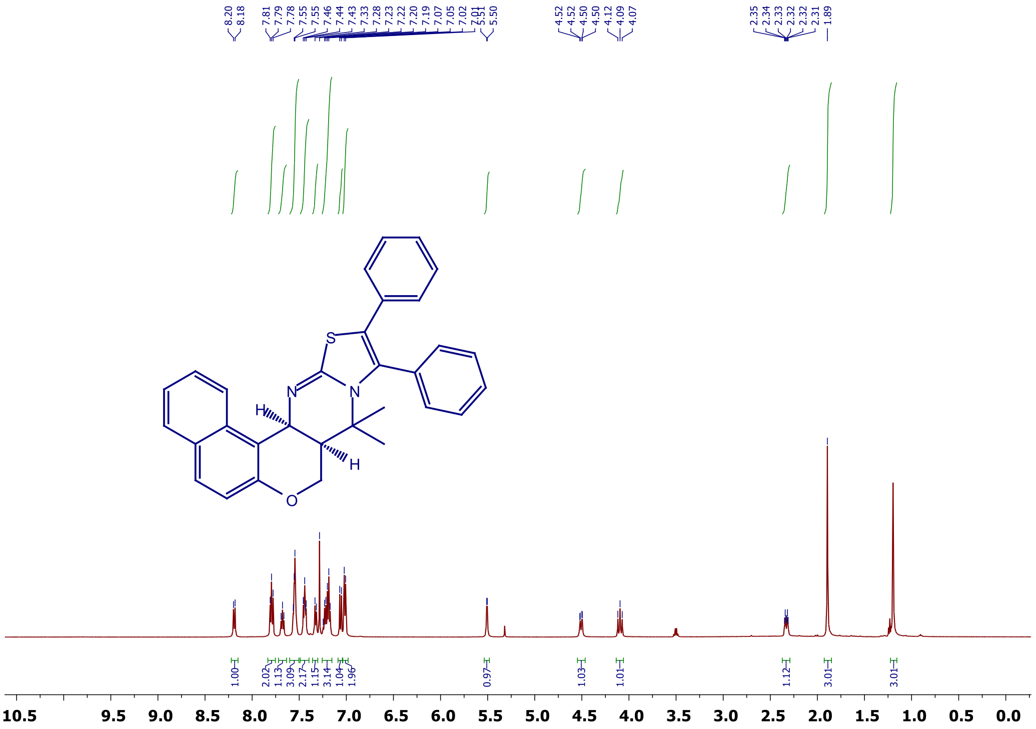

^{1}H NMR (500 MHz, $CDCl_3$) spectra of (8aS,14aS)-9,9-dimethyl-11,12-diphenyl-8a,14a-dihydro-8H,9H-benzo[5,6]chromeno[4,3-d]thiazolo[3,2-a]pyrimidine (**SIMR-1281**)

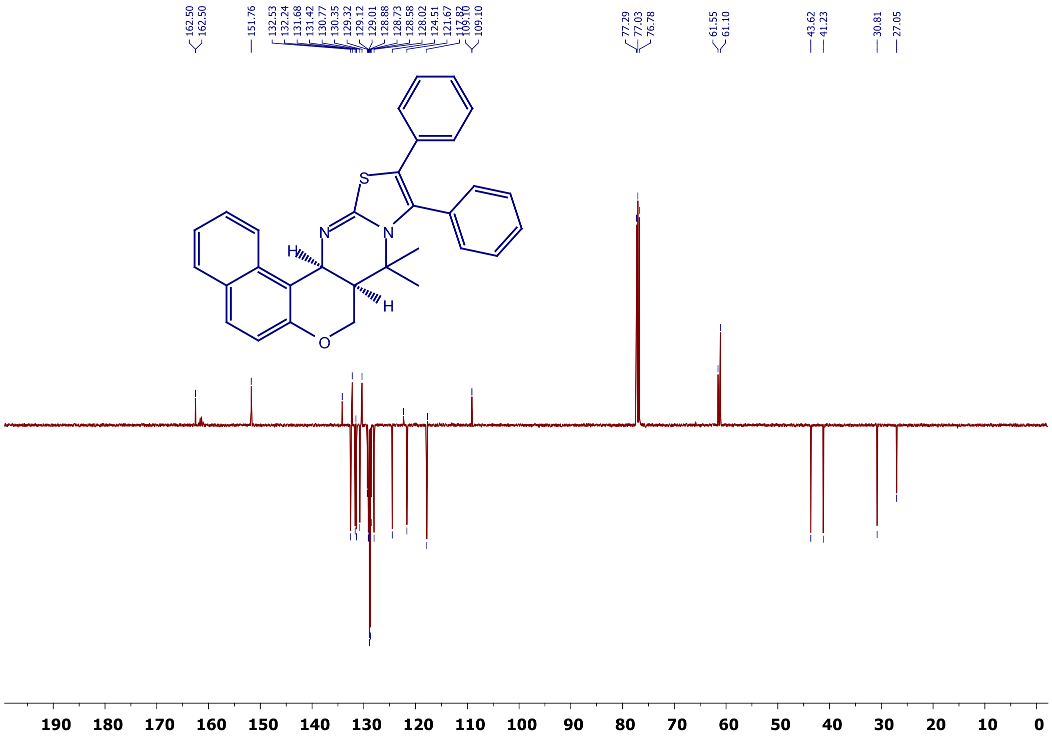

APT NMR (125 MHz, $CDCl_3$) spectra of (8aS,14aS)-9,9-dimethyl-11,12-diphenyl-8a,14a-dihydro-8H,9H-benzo[5,6]chromeno[4,3-d]thiazolo[3,2-a]pyrimidine (**SIMR-1281**)

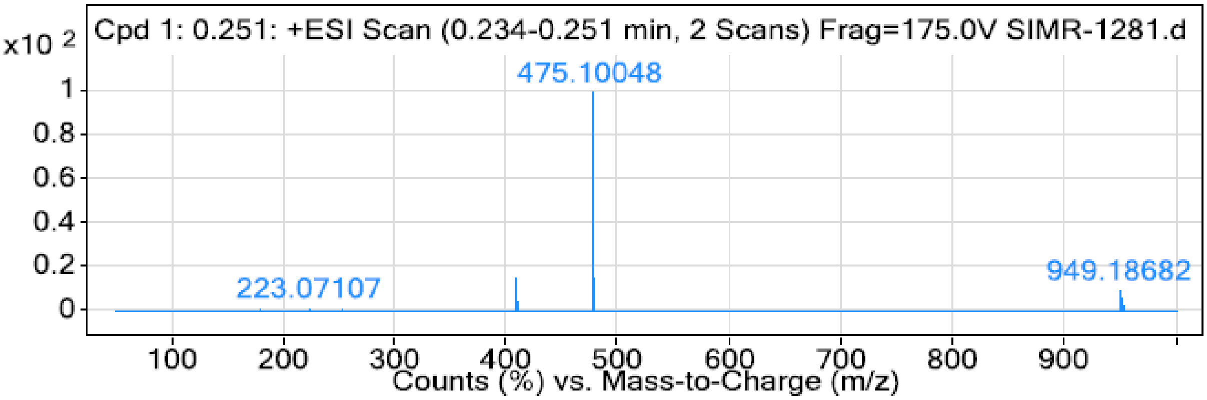

HRMS spectra of (8aS,14aS)-9,9-dimethyl-11,12-diphenyl-8a,14a-dihydro-8H,9H-benzo[5,6]chromeno[4,3-d]thiazolo[3,2-a]pyrimidine (**SIMR-1281**)

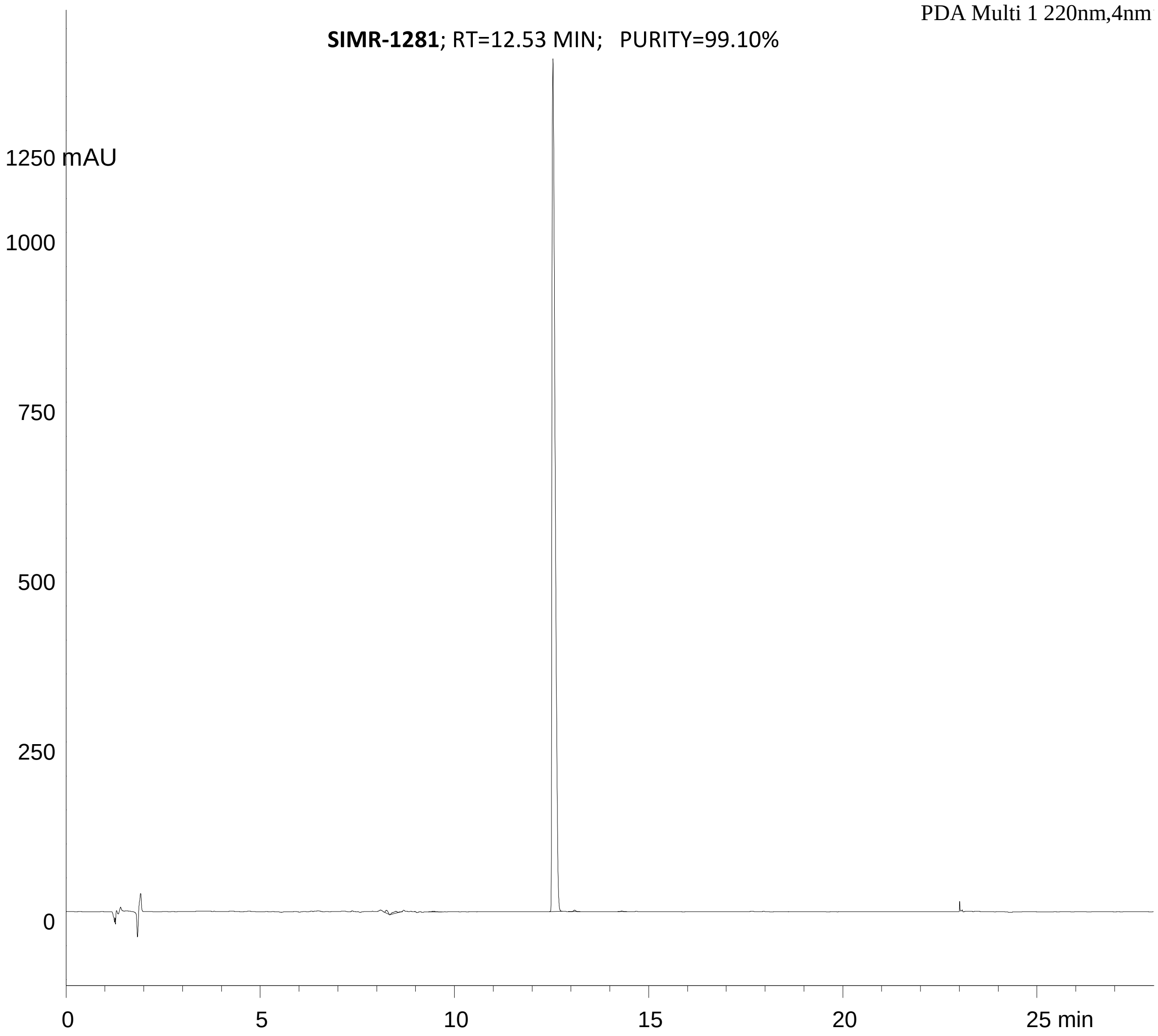

HPLC chromatograph of (8aS,14aS)-9,9-dimethyl-11,12-diphenyl-8a,14a-dihydro-8H,9H-benzo[5,6]chromeno[4,3-d]thiazolo[3,2-a]pyrimidine (**SIMR-1281**)
