# Supplementary material for: A Novel Benzopyrane Derivative Targeting Cancer Cell Metabolic and Survival Pathways
Source: Cancers (Basel). 2021 Jun 7;13(11):2840. doi: 10.3390/cancers13112840 (PMC8201054; doi:10.3390/cancers13112840)
Supplement: Supplementary file 1 [file cancers-13-02840-s001.zip › supplementary material/file S2.pdf]

A Novel benzopyrane derivative targeting cancer cell metabolic and survival pathways

Dana M. Zaher[1,2,#], Wafaa S. Ramadan[1,2,#], Raafat El-Awady[1,3], Hany A. Omar[1,3,4], Fatema Hersi[1,2], Vunnam Srinivasulu[1], Ibrahim Y. Hachim[1,2], Farah I. Almarzooq[1,5], Cijo G. Vazhappilly[1,6], Salim Merali[7], Carmen Merali[7], Nelson C. Soares[1,3], Paul Schilf [8], Saleh M. Ibrahim[1,2,8], Taleb H. Al-Tel[1,3,*]

*Correspondance: taltal@sharjah.ac.ae, Tel: +97165057417

# These coauthors contributed equally to this work and are both first authors

Supplementary file S2.

The MS/MS spectra of assigned to the identified peptides for each reported protein. Images were obtained from Maxquant viewer version (1.6.10.43)

sp|Q9NPH2|INO1_HUMAN Inositol-3-phosphate synthase 1

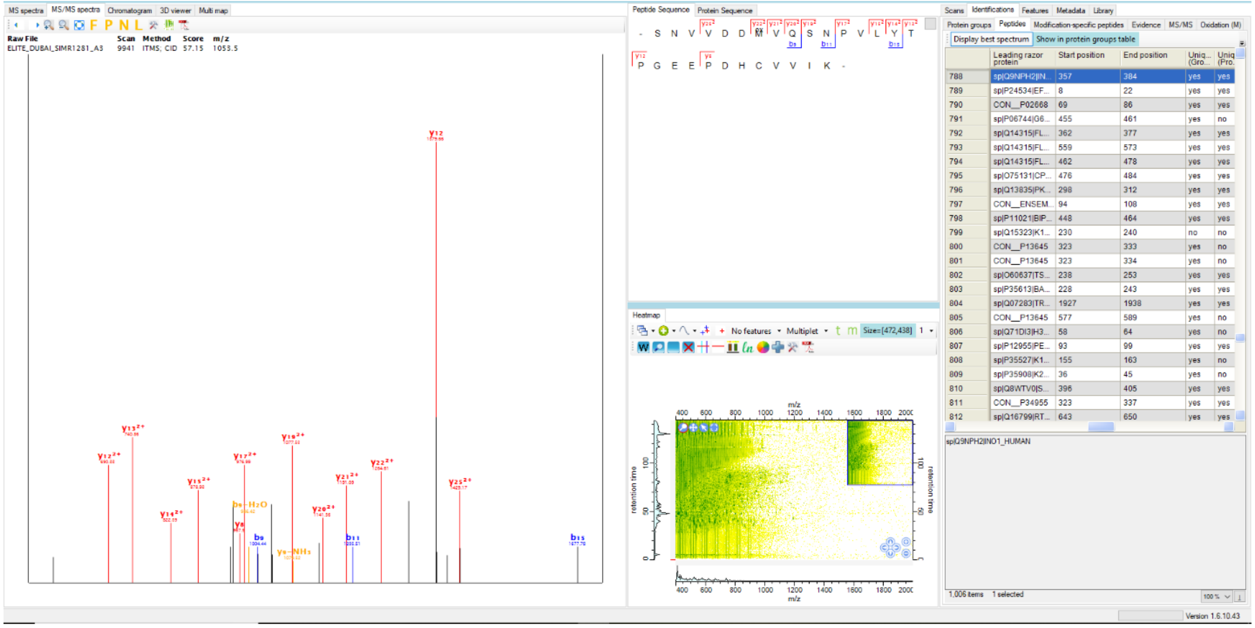

MS/MS fragmentation spectrum show both b- and y-ions of a peptide (**SNVVDDMVQSNPVLYTPGEEPDHCVVIK**) assigned to **sp|Q9NPH2|INO1_HUMAN Inositol-3-phosphate synthase 1**

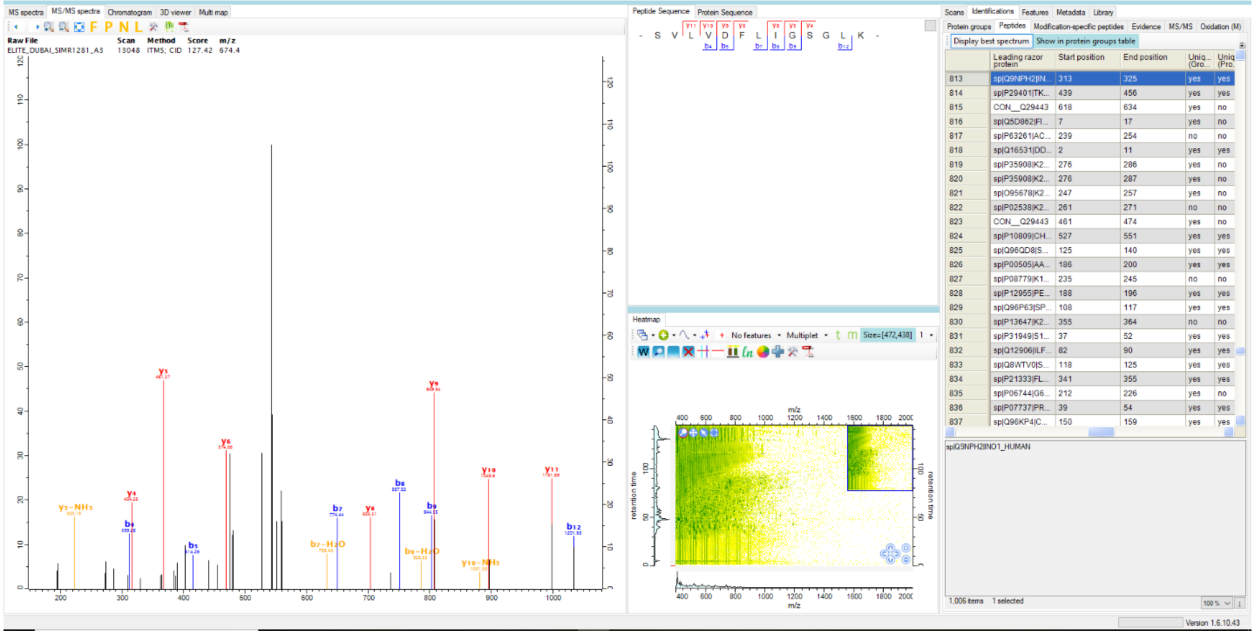

MS/MS fragmentation spectrum show both b- and y-ions of a peptide (**SVLVDFLIGSGLK**) assigned to **sp|Q9NPH2|INO1_HUMAN Inositol-3-phosphate synthase 1**

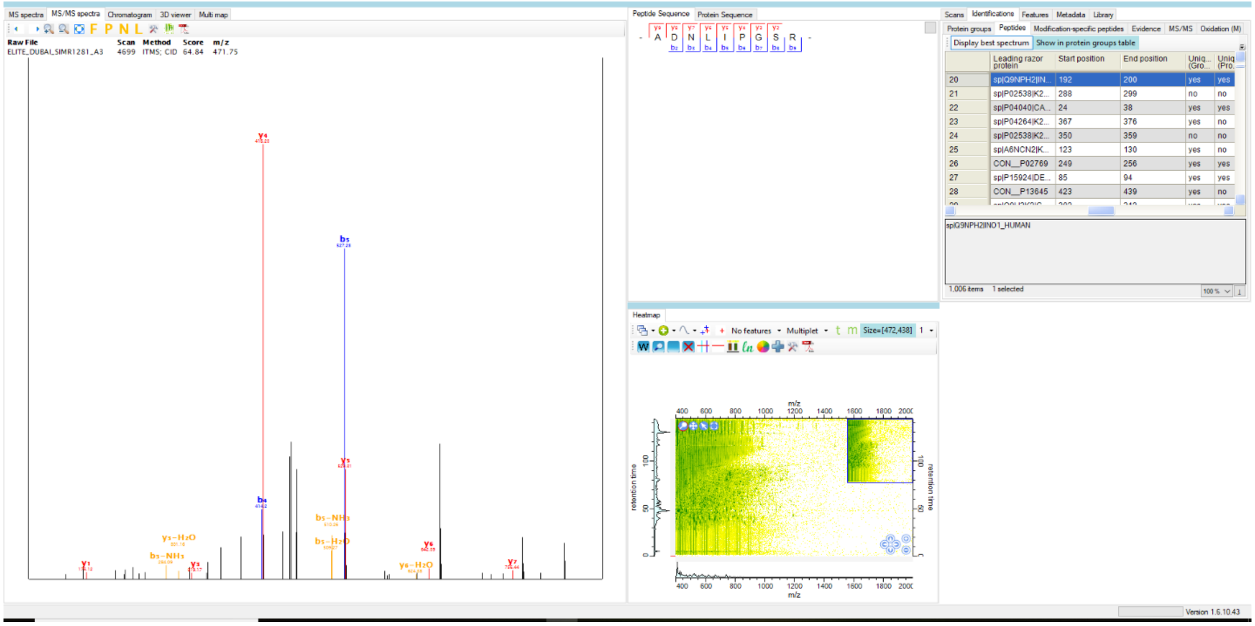

MS/MS fragmentation spectrum show both b- and y-ions of a peptide (**ADNLIPGSR**) assigned to **sp|Q9NPH2|INO1_HUMAN Inositol-3-phosphate synthase 1**

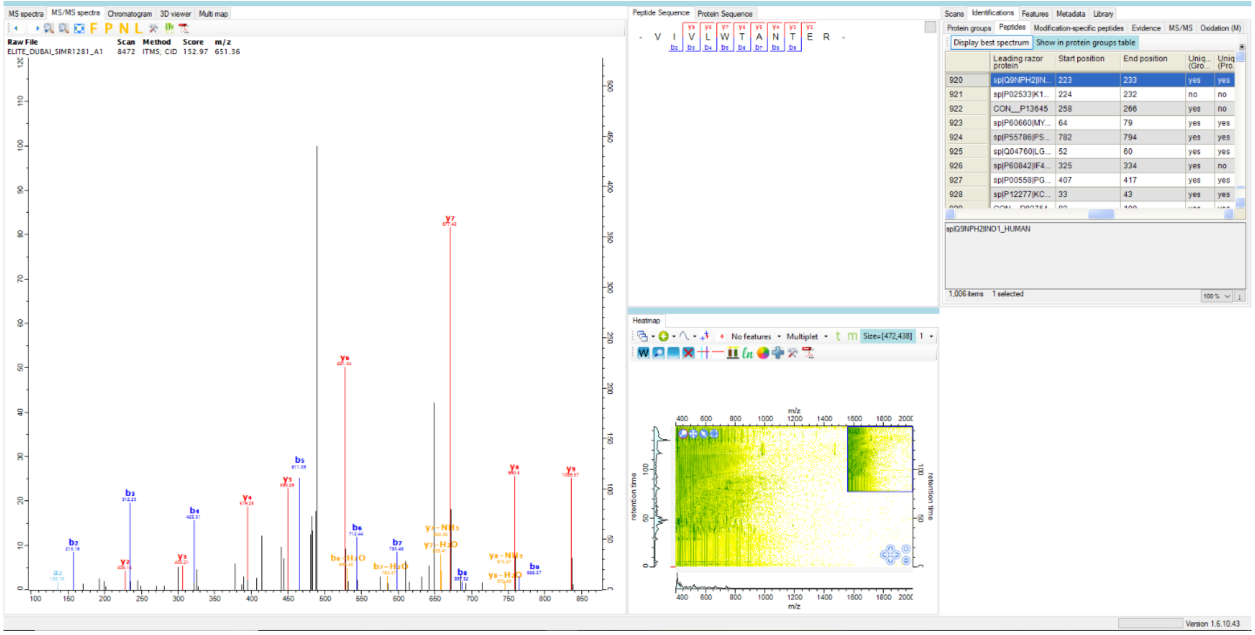

MS/MS fragmentation spectrum show both b- and y-ions of a peptide (**VIVLWTANTER**) assigned to **sp|Q9NPH2|INO1_HUMAN Inositol-3-phosphate synthase 1**

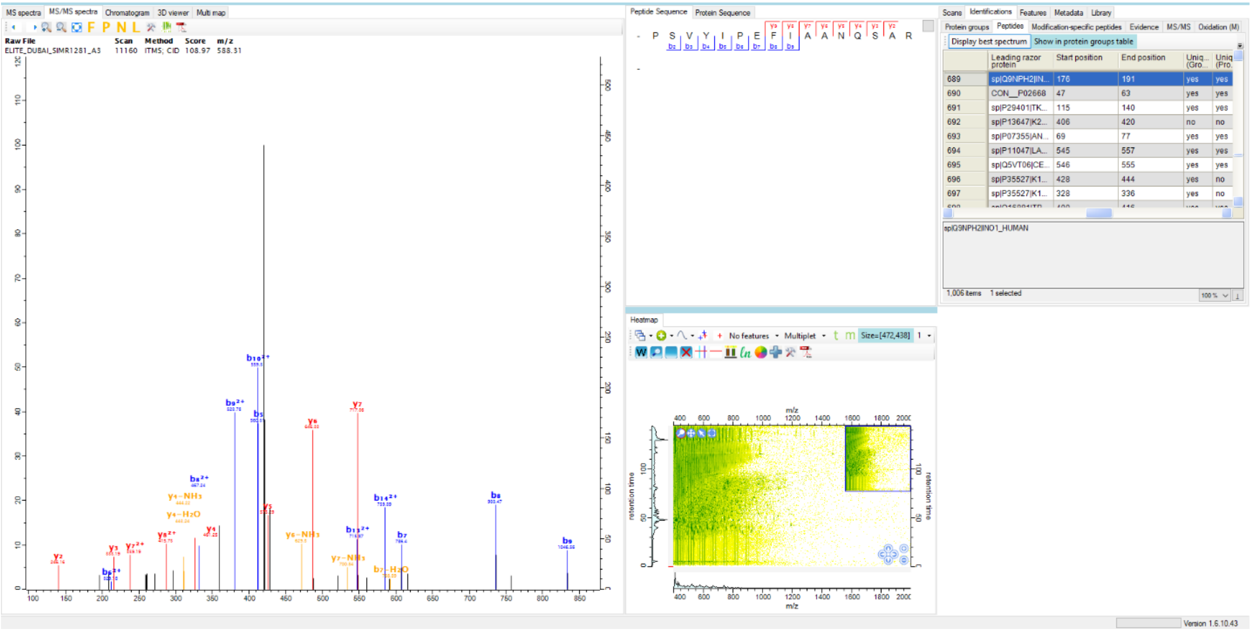

MS/MS fragmentation spectrum show both b- and y-ions of a peptide (**PSVYIPEFIAANQSAR**) assigned to **sp|Q9NPH2|INO1_HUMAN Inositol-3-phosphate synthase 1**

sp|P00390|GSHR_HUMAN Glutathione reductase 1, mitochondrial

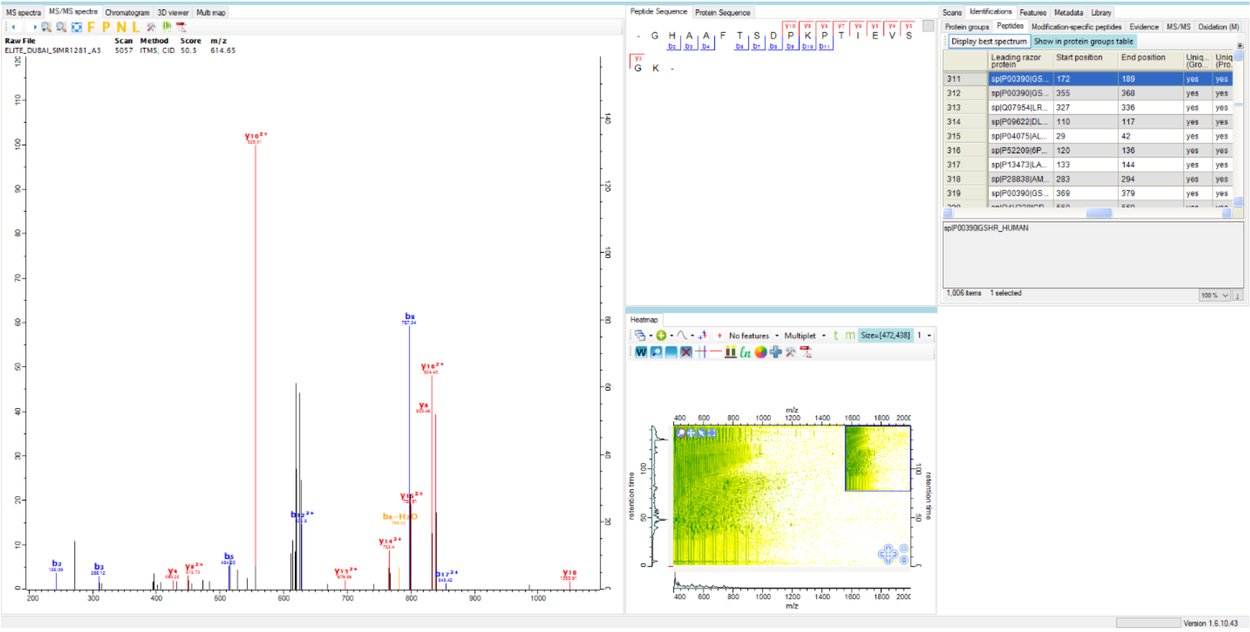

MS/MS fragmentation spectrum show both b- and y-ions of a peptide (**GHAAFTSDPKPTIEVS**) assigned to **sp|P00390|GSHR_HUMAN Glutathione reductase 1, mitochondrial**

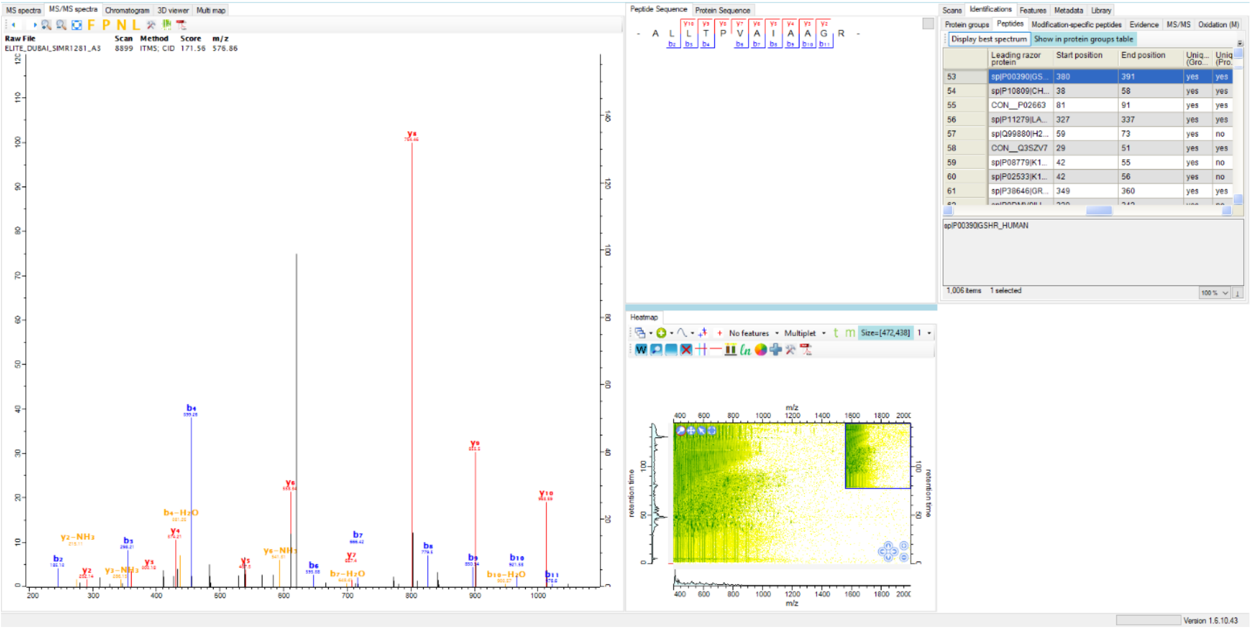

MS/MS fragmentation spectrum show both b- and y-ions of a peptide (**ALLTPVAIAAGR**) assigned to **sp|P00390|GSHR_HUMAN Glutathione reductase 1, mitochondrial**

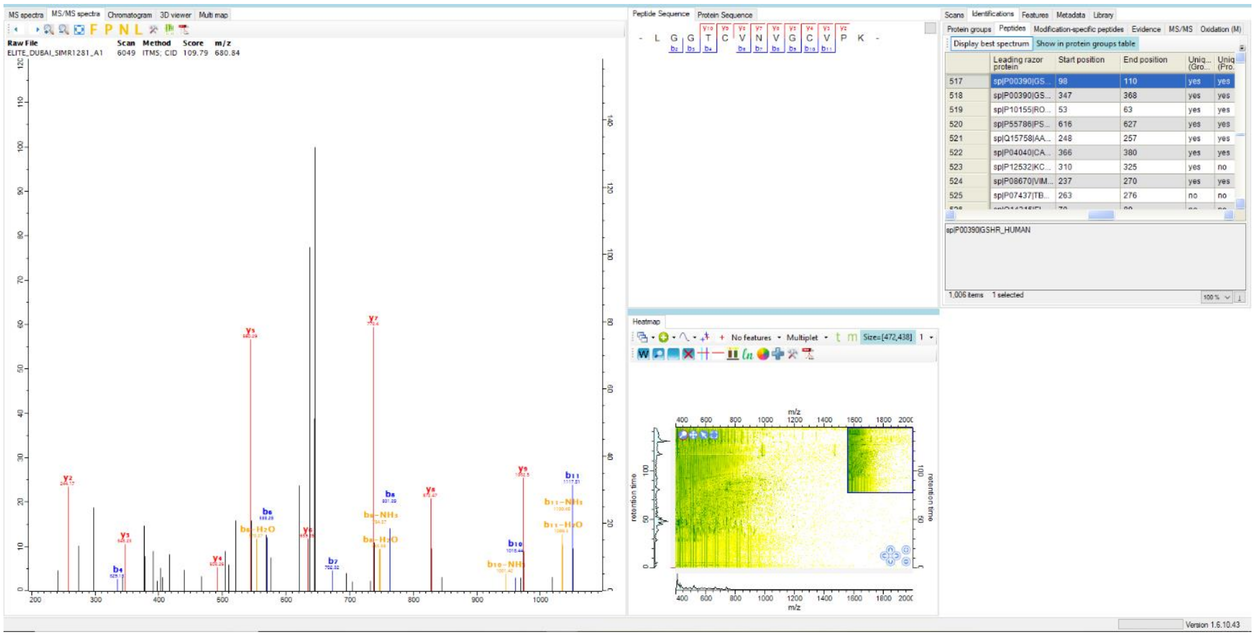

MS/MS fragmentation spectrum show both b- and y-ions of a peptide (**LGGTCVNVGCVPK**) assigned to **sp|P00390|GSHR_HUMAN Glutathione reductase 1, mitochondrial**

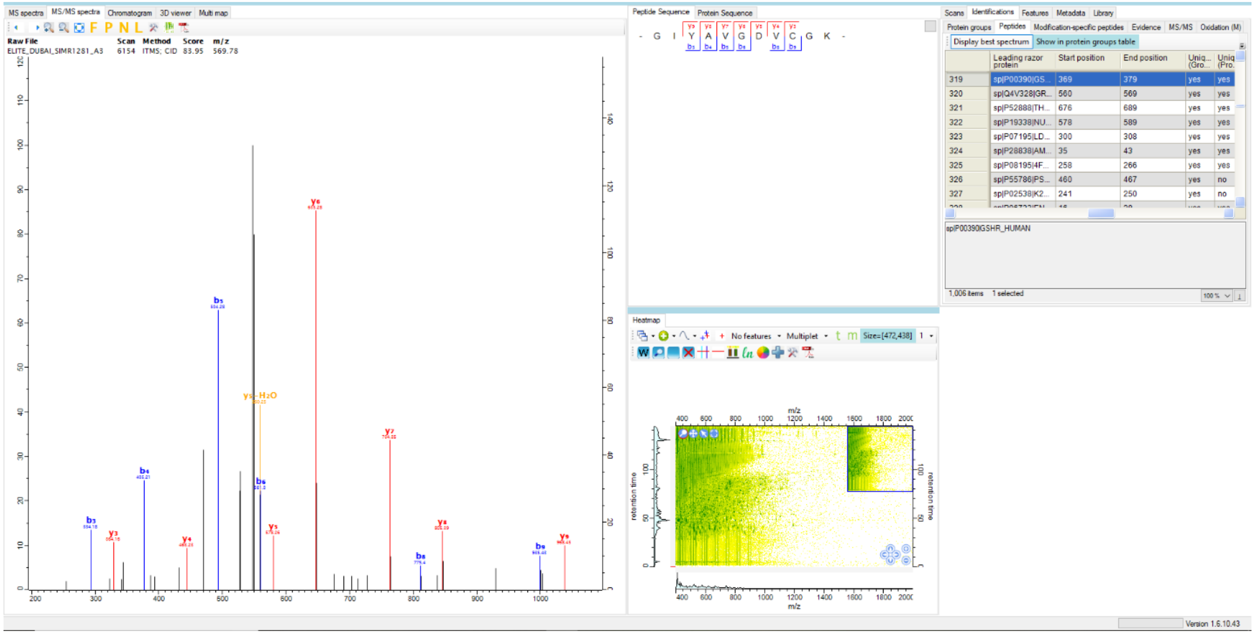

MS/MS fragmentation spectrum show both b- and y-ions of a peptide (**GIYAVGDCGK**) assigned to **sp|P00390|GSHR_HUMAN Glutathione reductase 1, mitochondrial**

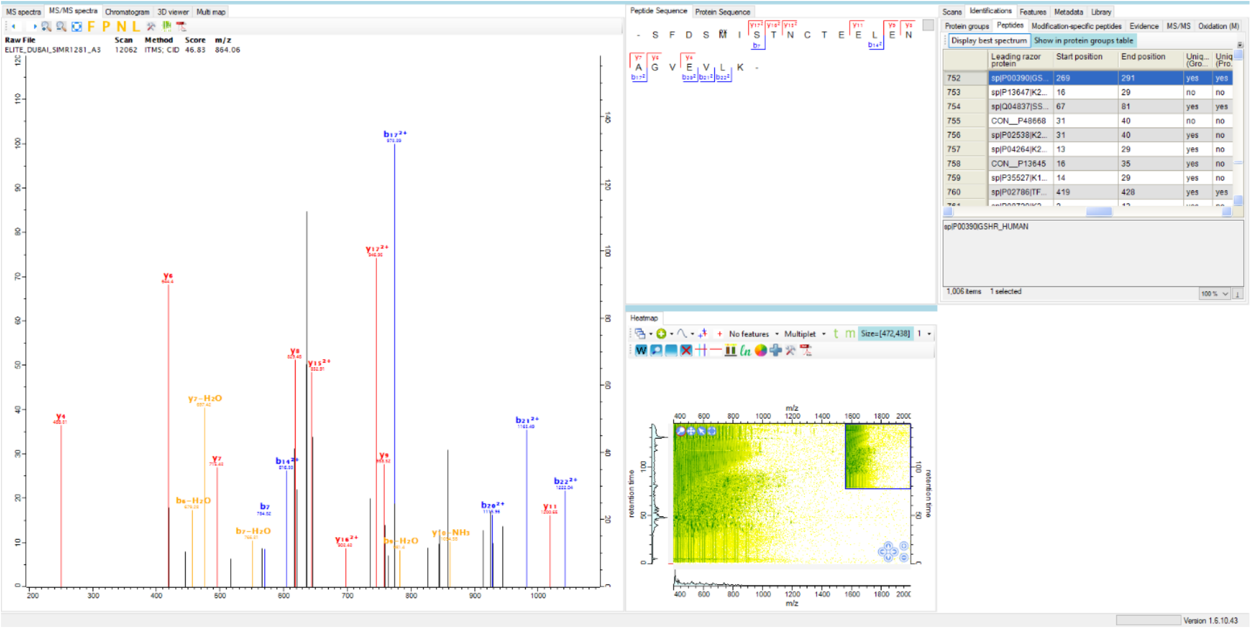

MS/MS fragmentation spectrum show both b- and y-ions of a peptide (**ALLTPVAIAAGR**) assigned to **sp|P00390|GSHR_HUMAN Glutathione reductase 1, mitochondrial**

sp|Q16881|TRXR1_HUMAN Thioredoxin reductase 1, cytoplasmic

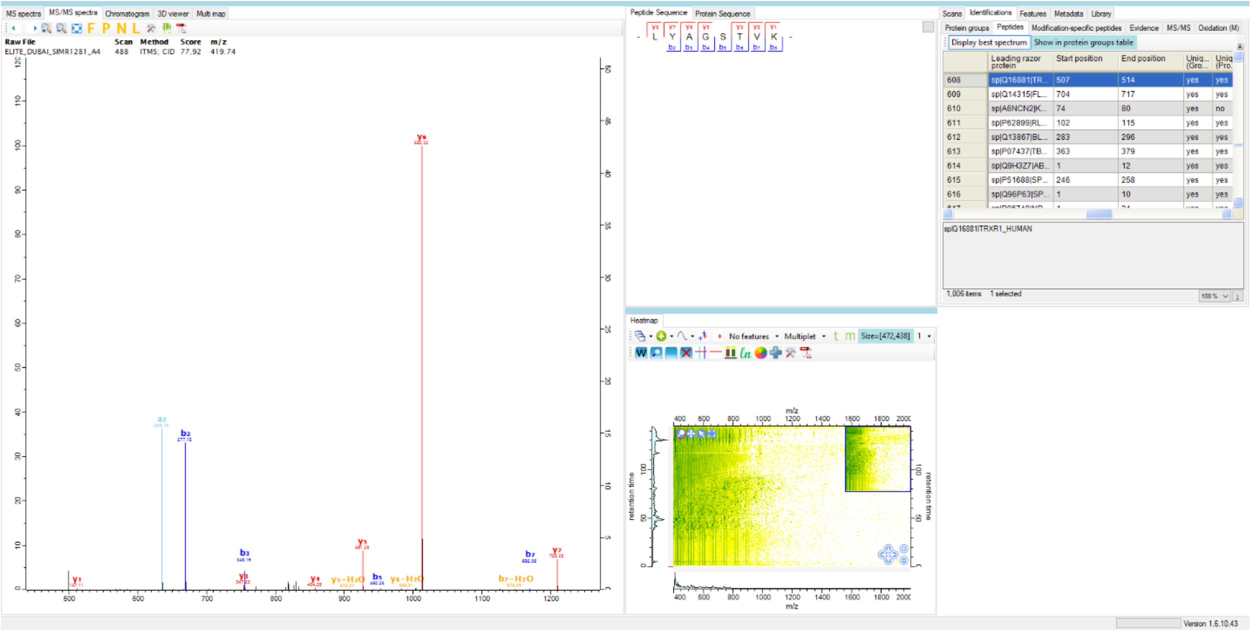

MS/MS fragmentation spectrum show both b- and y-ions of a peptide (**LYAGSTVK**) assigned to **sp|Q16881|TRXR1_HUMAN Thioredoxin reductase 1, cytoplasmic**

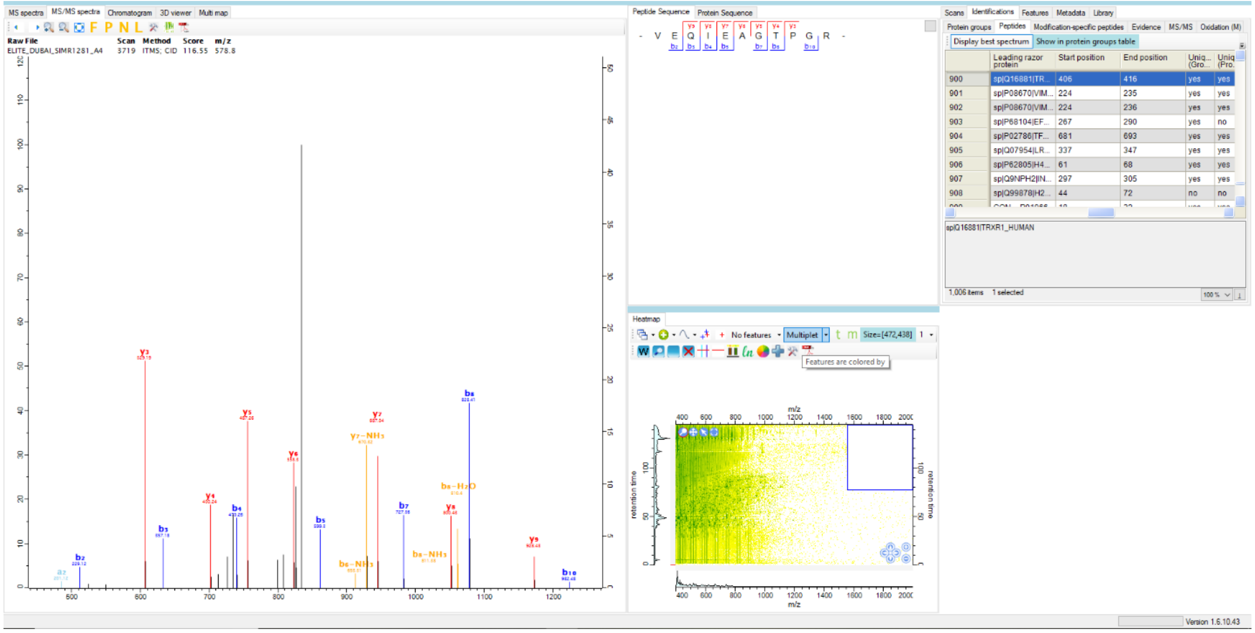

MS/MS fragmentation spectrum show both b- and y-ions of a peptide (**VEQIEAGTPGR**) assigned to **sp|Q16881|TRXR1_HUMAN Thioredoxin reductase 1, cytoplasmic**

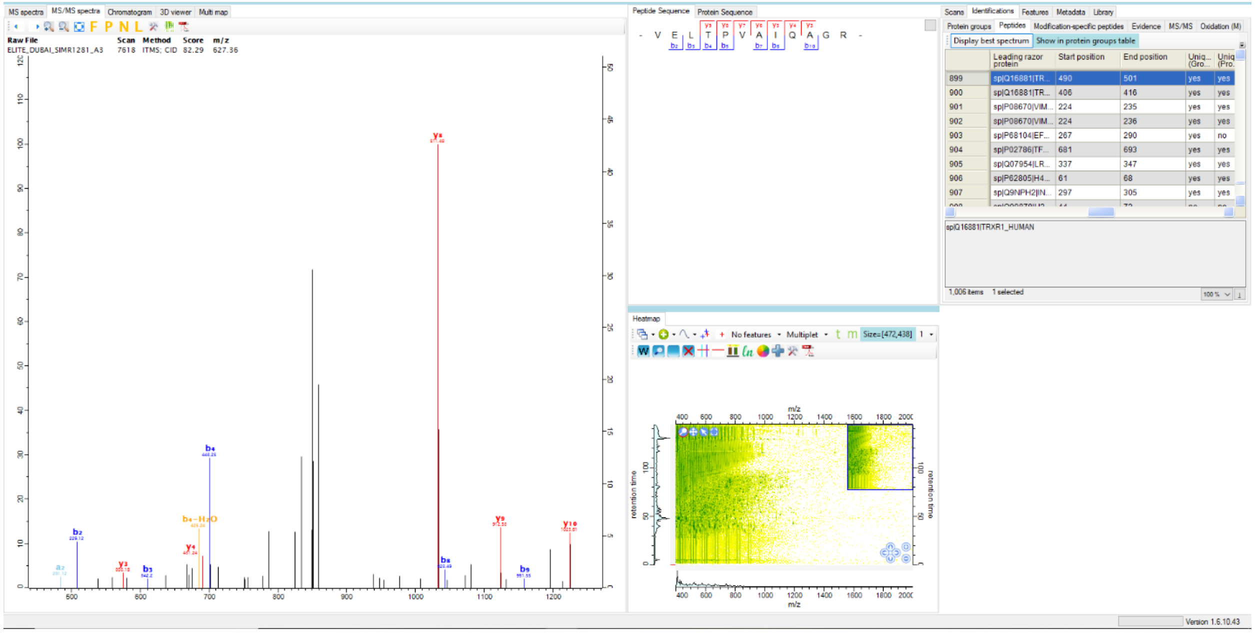

MS/MS fragmentation spectrum show both b- and y-ions of a peptide (**VELTPVAIQAGR**) assigned to **sp|Q16881|TRXR1_HUMAN Thioredoxin reductase 1, cytoplasmic**

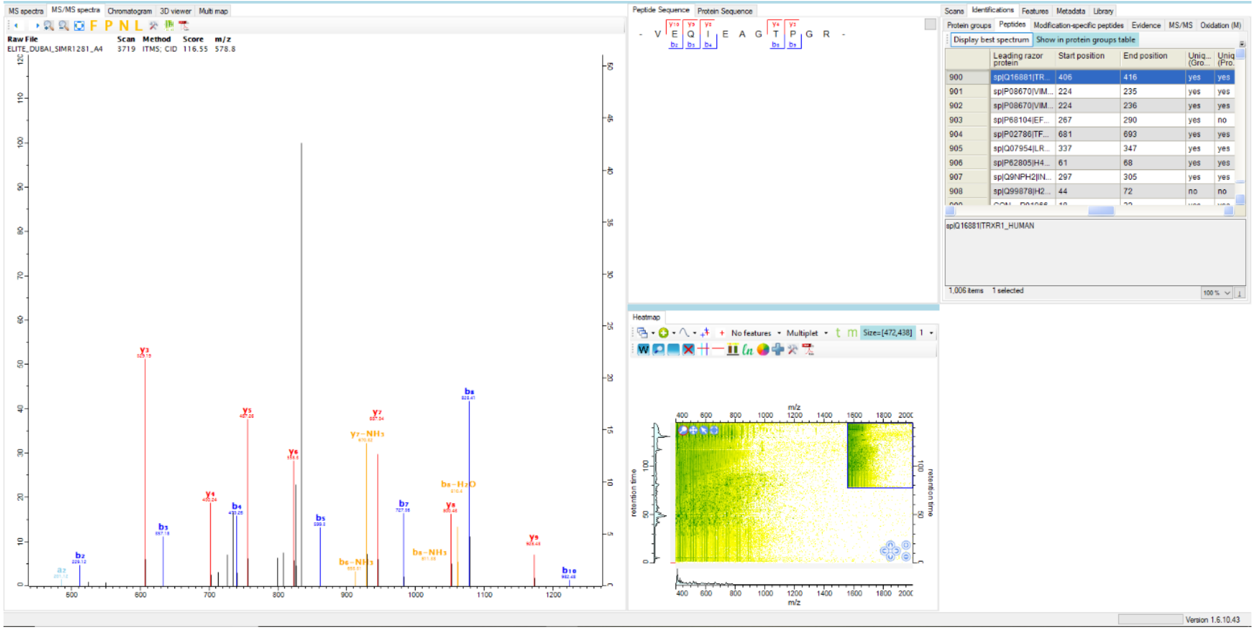

MS/MS fragmentation spectrum show both b- and y-ions of a peptide (**VEQIEAGTPGR**) assigned to **sp|Q16881|TRXR1_HUMAN Thioredoxin reductase 1, cytoplasmic**

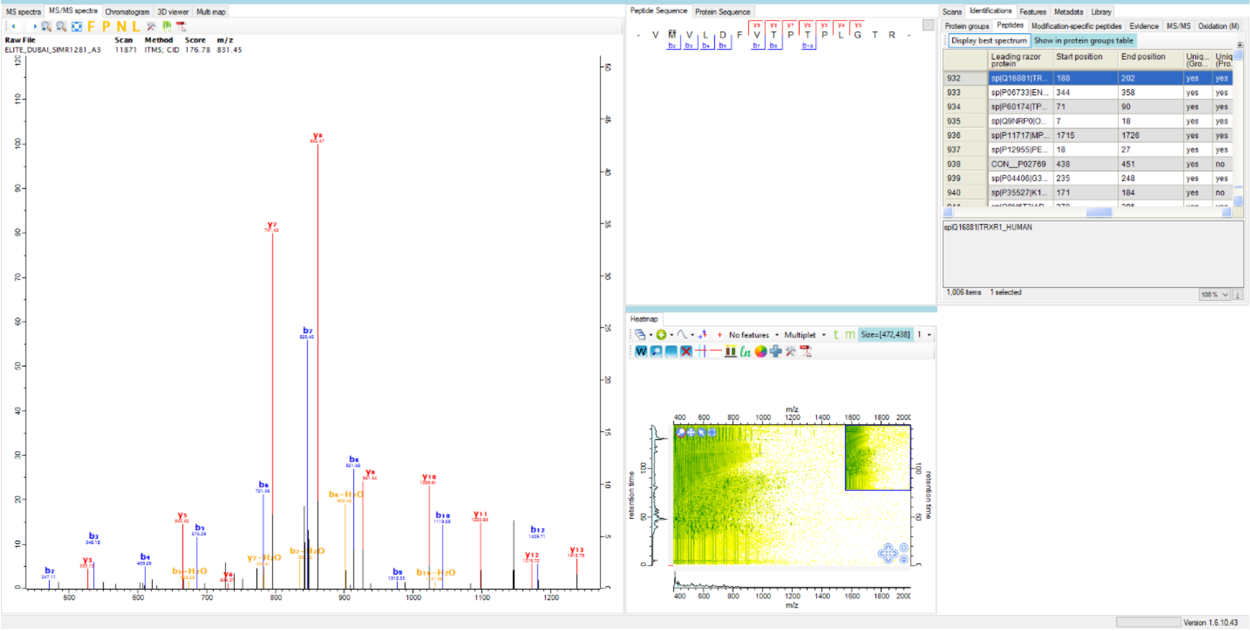

MS/MS fragmentation spectrum show both b- and y-ions of a peptide (**VMVLDFVTPTPLGTR**) assigned to **sp|Q16881|TRXR1_HUMAN Thioredoxin reductase 1, cytoplasmic**
